# Perinatal Care for Women with Foreign Citizenship in Trentino (North-East Italy): Retrospective Cohort Epidemiological Study

**DOI:** 10.3390/jcm15103704

**Published:** 2026-05-12

**Authors:** Riccardo Pertile, Stefania Poggianella, Fabrizio Taddei, Anna Rizzuto, Barbara Endrizzi, William Mantovani

**Affiliations:** 1 Department of Clinical and Evaluative Epidemiology, Trentino Integrated University Health Authority—ASUIT, 38123 Trento, Italy; anna.rizzuto@asuit.tn.it (A.R.); william.mantovani@asuit.tn.it (W.M.); 2 FBK Digital Health & Wellbeing Centre, 38123 Trento, Italy; spoggianella@fbk.eu; 3 Transmural Obstetric Gynecological Department, Trentino Integrated University Health Authority—ASUIT, 38123 Trento, Italy; fabrizio.taddei@asuit.tn.it (F.T.); barbara.endrizzi@asuit.tn.it (B.E.)

**Keywords:** women with foreign citizenship, perinatal health, maternal outcomes, neonatal outcomes, health assistance

## Abstract

**Background**: Foreign citizenship and low socioeconomic status are key determinants of health inequalities and may influence maternal and neonatal outcomes. This study aimed to assess maternal health during pregnancy and the main adverse maternal and neonatal outcomes related to labour and childbirth among women living in Trentino (Northern Italy), comparing women with Italian and foreign citizenship. **Methods**: A retrospective epidemiological study was conducted using data from the Birth Assistance Certificate (CedAP) database of the Autonomous Province of Trento. This study included all women who gave birth in Trentino between 2012 and 2016. Associations between citizenship and adverse outcomes were assessed using multivariable logistic regression models adjusted for potential confounders. **Results**: The analysis included 23,165 women, of whom 25.9% had foreign citizenship. Women with foreign citizenship showed a significantly higher risk of gestational diabetes mellitus compared with Italian women and an increased risk of extremely preterm birth (<28 weeks of gestation), particularly among women from Central and South America, Asia and Eastern Europe. Regarding labour and mode of delivery, women with foreign citizenship had a higher risk of caesarean section, especially among women from Central and South America and Africa. In terms of neonatal outcomes, infants born to women with foreign citizenship showed a higher likelihood of requiring phototherapy and admission to the neonatal intensive care unit. **Conclusions**: Significant differences were observed between immigrant and Italian women in both social determinants and maternal and neonatal perinatal outcomes. Identifying factors associated with adverse outcomes during pregnancy may help improve targeted maternal care and reduce health inequalities for both mothers and newborns.

## 1. Introduction

### 1.1. Statement of Significance

Issue: Citizenship and low socioeconomic status are fundamental determinants of health-related inequalities. Individuals belonging to the lower social classes are more likely to receive inadequate healthcare. These conditions can also impact maternal and neonatal outcomes.

What is Already Known: Women with foreign citizenship appear to be at greater risk of adverse maternal and neonatal outcomes, although the results in the literature are contradictory.

What this Paper Adds: Data reveal the presence of a different profile in terms of perinatal outcomes, both maternal and neonatal, between Italian women and women with foreign citizenship and show wide discrepancies within each ethnic group.

### 1.2. Background

The constant rise over the years in the migratory phenomenon has resulted in the need for hosting countries to deal with different populations in terms of both socio-demographic and clinical characteristics and cultural ones. These changes have also affected maternity support services. In Italy, 51% of the immigrant population consists of women, 56% of whom are of childbearing age [1,2].

Although the main perinatal outcomes among women with foreign citizenship have been extensively studied, the international literature shows conflicting results [3,4,5,6,7]. Some studies indicate immigrant status as a risk factor in terms of both maternal and neonatal outcomes.

As regards neonatal outcomes, what emerges is a higher rate of prematurity [8,9], low weight at birth [9,10], a low 5 min Apgar score [10], and both neonatal and perinatal mortality [11] between immigrant and native populations.

Turning to maternal health, a review [5] shows that women with foreign citizenship run a different risk (higher for women from Africa, Asia and South America and lower for women from Eastern Europe) of having a caesarean section than non-immigrant women. Other studies, meanwhile, report a higher risk for women with foreign citizenship of gestational diabetes mellitus (GDM) [10,12], preeclampsia and serious morbidity [13], emergency caesarean section [10], episiotomy and operative vaginal delivery [14], and maternal mortality [15].

Meanwhile, different studies have revealed that being an immigrant is not a risk factor associated with adverse perinatal outcomes, in terms of both preterm delivery [16] and macrosomia [10] and pregnancy-related hypertension [17], preeclampsia and induction of labour [10,14].

This discrepancy is described in the literature as “paradox of the healthy migrant”, a phenomenon linked to a possible migratory selection of healthy individuals, and is defined as the presence of healthcare outcomes equal to or even better than those in migrants, in spite of the unfavourable socioeconomic conditions [4,18,19]. The reasons underlying the increased risk of adverse outcomes reported in the literature are, instead, inadequate prenatal care, related to scant familiarity with or difficulty accessing the health services available in the area [18,20], as well as the presence of socio-cultural barriers [19,21,22].

Even the Italian literature reports that perinatal outcomes are worse among the newborns of immigrant mothers than among their Italian counterparts, especially in terms of preterm births [19,23], low 5 min Apgar scores, presence of respiratory diseases, need for intensive neonatal care [18], low weight at birth [19] and macrosomia [19,24]. Children born to women with foreign citizenship also show higher stillbirth rates than the newborns of Italian women [18,20].

Women with foreign citizenship, moreover, are seemingly more at risk of GDM [19] and having to face a post-term pregnancy [24], but less at risk of a caesarean section [19].

The Italian health system stipulates a universal cover for hospital care. A series of essential services, including maternity care, are offered free of charge to all immigrants, both legal and illegal [Legislative Decree n. 286/1998]. Despite that, it still seems difficult for the immigrant population to access services and healthcare [25].

Understanding whether inequalities exist in adverse maternal and perinatal outcomes is important for strengthening maternity management and support and for developing targeted intervention strategies during pregnancy and the preconception period.

After a first study about the termination of pregnancy (voluntary and spontaneous) [26], and a study on the care provided to women during gestation [27], the objective of this study is to describe and compare health status during pregnancy and the main adverse maternal and neonatal outcomes associated with labour and childbirth among women with Italian citizenship and women with foreign citizenship in Trentino, a region in Northeast Italy. Quantifying the phenomenon is relevant if we want to implement intervention strategies both when pregnancy is underway and in the pre-conception phase, with the aim of improving the maternal and foetal state of health. A secondary objective is to assess the maternal characteristics and health determinants (such as social factors and cultural conditions) that discriminate against foreign citizenship.

## 2. Methods

### 2.1. Study Design and Data Source

This retrospective population-based cohort study was conducted using data from the Birth Assistance Certificate (CedAP) registry of the Autonomous Province of Trento (Northern Italy). The CedAP is a standardised administrative database that prospectively collects information on maternal characteristics, pregnancy, delivery, and neonatal outcomes for all births occurring in the province, including hospital births, planned home births, and unplanned out-of-hospital births subsequently assisted by healthcare professionals. The study period ranged from 1 January 2012 to 31 December 2016.

This study is part of a broader analysis, called A MatCH For Women (Access to Maternal and Child Health Services by Foreign Women), which includes all women who had access to Trentino health services from 2012 to 2016, both for childbirth and a termination of pregnancy (spontaneous or voluntary). This timespan was selected to be able to assess the state of health of women during maternity and that of their children from birth to 6 years of life (2018–2022 five-year period).

Given the vast quantity of data collected and analysed, the results have been published in various studies, depending on the maternal and infant conditions examined [26,27].

This study was approved by the Ethics Committee of the Provincial Health Services (APSS) (approval number: 11115; 27 June 2022). The data were anonymised prior to analysis. This study was conducted in accordance with the Declaration of Helsinki.

### 2.2. Study Population

This study included all women who gave birth in the Province of Trento during the study period. Women with missing information on citizenship were excluded.

Women were categorised according to citizenship into the following:

- Italian citizens (reference group);
- Foreign citizens, further classified into five geographical macro-areas based on the Italian National Institute of Statistics (ISTAT) classification: Africa, Asia, Central and South America, Eastern Europe, and other countries (including EU countries and other high-income countries) [28].

### 2.3. Variables and Outcomes

Exposure: The main exposure variable was maternal citizenship, categorised as Italian citizenship and foreign citizenship.

Outcomes: Outcomes were grouped into three domains.

1. Pregnancy outcomes:

Gestational diabetes mellitus, diagnosed according to standard oral glucose tolerance test criteria.

Hypertensive disorders of pregnancy (gestational hypertension and preeclampsia).

Intrahepatic cholestasis of pregnancy (ICP), diagnosed based on clinical symptoms and elevated serum bile acids.

Group B streptococcus (GBS) colonisation, assessed by vaginal–rectal swab culture performed during pregnancy.

Syphilis, assessed by the Venereal Disease Research Laboratory (VDRL) test.

Hepatitis B infection, defined by hepatitis B surface antigen (HBsAg) positivity.

Hepatitis C infection, defined by anti-HCV antibody positivity.

Human immunodeficiency virus (HIV) infection, defined by positive antibody testing.

Preterm birth, categorised as moderate to late preterm (32–36 weeks), very preterm (28–31 weeks), and extremely preterm (<28 weeks).

Stillbirth (≥180 days of gestation) [29].

2. Labour and delivery outcomes:

Mode of delivery (vaginal delivery, caesarean section and assisted/operative vaginal delivery).

Induction of labour.

Epidural analgesia.

Episiotomy.

3. Neonatal outcomes:

Birth weight (<2500 g; ≥4500 g) [30].

Five min Apgar score ≤ 3.

Neonatal resuscitation (oxygen/intubation).

Admission to the neonatal intensive care unit (NICU).

Phototherapy.

Length of hospital stay.

Type of feeding at discharge.

Definitions of key variables were based on ICD-9-CM codes and national guidelines.

Covariates:

Potential confounders included maternal age, parity, marital status, educational level, employment status, smoking during pregnancy, and multiple pregnancy. Additional clinical covariates (e.g., maternal comorbidities, gestational age, and foetal conditions) were included depending on the specific outcome analysed.

Covariates were selected a priori based on clinical relevance and the previous literature on perinatal outcomes. All selected covariates were included in the multivariable models.

### 2.4. Statistical Analyses

Descriptive statistics were used to summarise maternal characteristics and outcomes. Continuous variables were reported as means and standard deviations (SDs) or medians and interquartile ranges (IQRs), as appropriate. Categorical variables were expressed as frequencies and percentages. Group comparisons were performed using the chi-square test for categorical variables and Student’s *t*-test or the Mann–Whitney U test for continuous variables.

Associations between citizenship and outcomes were assessed using univariable and multivariable logistic regression models. The results were reported as odds ratios (ORs) with 95% confidence intervals (95% CIs). Separate multivariable models were fitted for each outcome. The covariates included in each model are specified in the corresponding tables.

Multicollinearity among covariates was assessed using variance inflation factors (VIFs), and no significant collinearity was detected.

Missing data were minimal (<2%) and handled using complete-case analysis.

All analyses were performed using Stata version 17 (StataCorp, College Station, TX, USA).

This study was reported in accordance with the STROBE guidelines.

## 3. Results

### 3.1. Socio-Demographic Characteristics

This study included 23,165 women who delivered in Trentino between 2012 and 2016, after excluding 13 cases with missing citizenship data. Women with foreign citizenship accounted for 25.9% of the population, mainly originating from Eastern Europe, followed by Africa and Asia. A total of 23,600 newborns were included in neonatal analyses.

As shown in Table 1, women with foreign citizenship differed substantially from Italian women in several socio-demographic characteristics. They were younger at delivery and more frequently multiparous, whereas Italian women were more often nulliparous. Women with foreign citizenship also showed a lower educational level and a markedly lower employment rate.

In addition, women with foreign citizenship were less likely to smoke during pregnancy and were more frequently married, while no differences were observed in multiple pregnancies.

### 3.2. Adverse Outcomes

Table 2 summarises maternal and neonatal outcomes. Overall, differences between the two groups were selective rather than widespread.

Women with foreign citizenship showed a higher prevalence of metabolic and infectious conditions, particularly GDM and positive serological tests (including syphilis, hepatitis B, hepatitis C and HIV). They also experienced worse neonatal outcomes, including a greater need for neonatal resuscitation with oxygen and a higher rate of admission to neonatal intensive care.

Conversely, some conditions were more frequent among Italian women, including hypothyroidism, ICP, and the use of epidural analgesia. Italian women were also less likely to exclusively breastfeed at discharge.

No significant differences were observed for several major outcomes, including hypertensive disorders in pregnancy, preterm birth, stillbirth, mode of delivery, birthweight, and 5 min Apgar score.

### 3.3. Multivariable Analysis

Multivariable logistic regression analyses (Table 3, Table 4 and Table 5) confirmed these patterns after adjustment for potential confounders.

Women with foreign citizenship had a markedly increased risk of infectious diseases, particularly syphilis and hepatitis B, whereas no significant differences were found for hepatitis C and HIV infection.

When stratified by area of origin, the risk of syphilis was highest among women from Central and South America, Eastern Europe, and Africa. Similarly, the highest risk of hepatitis B was observed among women from Eastern Europe, Asia, and Africa.

### 3.4. Outcomes in Pregnancy

After excluding women with pre-existing conditions, multivariable analysis confirmed that women with foreign citizenship had a significantly higher risk of GDM compared with Italian women. This risk was particularly pronounced among women from Asia and Africa, whereas it was lower in other geographic groups and lowest among Italian women.

Women with foreign citizenship also showed an increased risk of extremely preterm birth. However, given the very low incidence of this outcome, these findings should be interpreted with caution.

Conversely, Italian women had a higher risk of ICP, although this condition was relatively rare in both groups.

### 3.5. Outcomes in Labour/Childbirth

Multivariable analysis indicated that women with foreign citizenship had an overall higher likelihood of caesarean section compared with Italian women, while no differences were observed for assisted/operative vaginal delivery.

When stratified by area of origin, the risk of caesarean section was higher among women from Central and South America and Africa, whereas it was lower among women from Eastern Europe and comparable in other groups.

No significant differences were observed between Italian women and women with foreign citizenship in terms of labour induction, episiotomy, or use of epidural analgesia.

### 3.6. Neonatal Outcomes

Regarding neonatal outcomes (Table 5), women with foreign citizenship had a higher adjusted risk of NICU admission and need for phototherapy.

In contrast, the previously observed higher rate of neonatal resuscitation with oxygen among women with foreign citizenship was no longer significant after adjustment for confounders.

No association was found between citizenship and 5 min Apgar score ≤ 3, a finding consistent with the absence of differences in mean Apgar scores across groups.

Finally, Italian women were more likely to use formula feeding at discharge compared with women with foreign citizenship.

## 4. Discussion

Our results reveal a different profile in terms of perinatal outcomes, both maternal and neonatal, between Italian women and women with foreign citizenship for some of the investigated outcomes. This finding, based on what emerges from the literature, might be due to a higher incidence of maternal diseases in the population of women with foreign citizenship (also highlighted by our study with regard to GDM, anaemia and infectious diseases) [18,31], while other factors (e.g., genetic differences) cannot be assessed within the scope of the present study. Furthermore, social exclusion, worse living conditions, and less recourse to prenatal care, all factors more frequently encountered among women with foreign citizenship, can partly contribute to explaining these differences [31,32,33].

As for outcomes during pregnancy, it emerges that the immigrant population is at a higher risk of suffering from GDM than Italian women, as identified in other studies [10,12].

In relation to preterm birth, our study highlights a higher risk in women with foreign citizenship solely as regards extremely preterm birth, especially in the population of women from Central and South America, Asia and Eastern Europe. These data are partly in conflict with what emerges from the literature [3,18,19], which identifies different ethnic groups as more at risk of preterm birth (particularly African women).

Meanwhile, women with foreign citizenship were found to be less exposed to the risk of pregnancy-related cholestasis than their Italian counterparts. This phenomenon has hardly been researched in the literature and requires more in-depth evaluation.

Among outcomes assessed during labour/childbirth, women with foreign citizenship proved to be exposed to a higher risk of caesarean section than Italian women. More specifically, the risk of undergoing a caesarean section is significantly higher in women from Central and South America and from Africa, and significantly lower in women from Eastern Europe. We find conflicting data in the literature on this issue [5], as well as on the increased risk for women with foreign citizenship of an assisted/operative vaginal delivery [14], data not confirmed by our analysis.

Lastly, considering neonatal outcomes, we found a statistically significant risk for women with foreign citizenship of having a newborn who requires phototherapy (data hitherto inadequately investigated in the literature) or having to be admitted to the NICU. This outcome, too, is the subject of intense debate, which means that we have come across studies that confirm an increased risk for women with foreign citizenship [18] and others that do not reveal any differences in this regard between immigrant and resident populations [14].

Women with foreign citizenship also appear to be at lower risk of breastfeeding their infants with formula on discharge. This phenomenon is consistent with what was reported by a recent systematic review [34], according to which women with foreign citizenship achieve better results in breastfeeding than their non-immigrant counterparts, although the reasons are still hardly known.

The heterogeneity we find in the literature is likely to be rooted in the different types of ethnicities investigated and the cumulative effect of social, economic and cultural factors that eschew easy assessment. However, given the impact such conditions could have on maternal–neonatal health, it is important to maintain a particular level of attention on women with foreign citizenship with a view to providing the best possible assistance.

Overall, our findings do not clearly support the “healthy migrant paradox,” as women with foreign citizenship exhibited an increased risk for several adverse outcomes, such as GDM or NICU admission. At the same time, the heterogeneity across subgroups highlights the importance of avoiding oversimplified interpretations of migrant health.

### Limitations and Strengths of This Study

This study has several strengths. The birth support certificate (Cedap) used in the maternity facilities of the province of Trento collects numerous data prospectively. The data collection system is standardised throughout the provincial territory, while variables are gathered with a very high degree of completeness and checked for accuracy and quality against the corresponding data from medical records. Lastly, the sample taken into consideration is quite broad and representative of the population.

A key limitation of this study is the lack of direct socioeconomic indicators. Although proxy variables such as education and employment were included, residual confounding is likely. Socioeconomic disadvantage, social exclusion, and barriers to healthcare access may substantially contribute to the observed disparities. It is plausible that, within the group of women with foreign citizenship, some differences are due to integration and cultural levels, as well as to economic levels. The condition of immigrant women was defined in relation to citizenship, without being able to probe the overall situation more in depth.

## 5. Conclusions

This analysis evinces the existence of differences between immigrant and Italian women in terms of both social determinants and maternal or neonatal perinatal outcomes. These outcomes, however, show wide discrepancies within each ethnic group.

In order to ensure better healthcare for both mothers and newborns, the factors most capable of affecting the risk of adverse outcomes should be identified during pregnancy. Account must accordingly be taken of the cultural and socioeconomic differences that characterise migrant women and their families.

## Figures and Tables

**Table 1:** Socio-demographic characteristics of the study population stratified by Italian and foreign citizenship (2012–2016).

	Total (N = 23,165)	Italian Women (N = 17,177)	Women with Foreign Citizenship (N = 5988)	*p*-Value
**Maternal age (years), categories**				<0.001
≤17	47 (0.2%)	31 (0.2%)	16 (0.3%)	
18–23	1666 (7.2%)	736 (4.3%)	930 (15.5%)	
24–29	6224 (26.9%)	3963 (23.1%)	2261 (37.8%)	
30–35	9177 (39.6%)	7271 (42.3%)	1906 (31.8%)	
36–41	5367 (23.2%)	4574 (26.6%)	793 (13.2%)	
≥42	684 (3.0%)	602 (3.5%)	82 (1.4%)	
Maternal age (years)	31.7 ± 5.5	32.6 ± 5.2	29.3 ± 5.5	<0.001
Median (IQR)	32.0 (28.0–36.0)	33.0 (29.0–36.0)	29.0 (25.0–33.0)	
**Parity**				<0.001
Nulliparous	10,903 (47.1%)	8430 (49.1%)	2473 (41.3%)	
Primiparous	8633 (37.3%)	6489 (37.8%)	2144 (35.8%)	
Multiparous	3554 (15.3%)	2211 (12.9%)	1343 (22.4%)	
Grand multiparous (≥5)	75 (0.3%)	47 (0.3%)	28 (0.5%)	
**Marital status**				<0.001
Unmarried	6962 (30.1%)	5967 (34.7%)	995 (16.6%)	
Married	15,484 (66.8%)	10,652 (62.0%)	4832 (80.7%)	
Divorced/separated/widowed	719 (3.1%)	558 (3.2%)	161 (2.7%)	
**Education level**				<0.001
Primary education or less	523 (2.3%)	62 (0.4%)	461 (7.7%)	
Middle school	3226 (13.9%)	1652 (9.6%)	1574 (26.3%)	
High school or higher	19,416 (83.8%)	15,463 (90.0%)	3953 (66.0%)	
**Professional condition**				<0.001
Employed	15,101 (65.2%)	13,362 (77.8%)	1739 (29.0%)	
Unemployed	2807 (12.1%)	1789 (10.4%)	1018 (17.0%)	
Other	5257 (22.7%)	2026 (11.8%)	3231 (54.0%)	
**Pregnancy**				0.076
Singleton	22,742 (98.2%)	16,846 (98.1%)	5896 (98.5%)	
Twin	413 (1.8%)	325 (1.9%)	88 (1.5%)	
Triplet	11 (0.0%)	7 (0.0%)	4 (0.1%)	
**Smoking during pregnancy**	1437 (6.2%)	1128 (6.6%)	309 (5.2%)	<0.001

Statistical significance was assessed using the chi-square test or Fisher’s exact test, and the Wilcoxon–Mann–Whitney test for continuous variables.

**Table 2:** Proportion (%) of maternal and neonatal adverse outcomes in the study population stratified by Italian and foreign citizenship (2012–2016).

	Total (N = 23,165) N (%)	Italian Women (N = 17,177) N (%)	Women with Foreign Citizenship (N = 5988) N (%)	*p*-Value
**Hypertensive disorders in pregnancy**				
Chronic hypertension	96 (0.4%)	65 (0.4%)	31 (0.5%)	0.15
Gestational hypertension	256 (1.1%)	203 (1.2%)	53 (0.9%)	0.06
**Preeclampsia**	220 (1.0%)	164 (1.0%)	56 (0.9%)	0.89
**Diabetes**				
Pre-existing diabetes mellitus	95 (0.4%)	62 (0.4%)	33 (0.6%)	0.05
GDM	1113 (4.8%)	663 (3.9%)	450 (7.5%)	<0.001
**Hypothyroidism**	1880 (8.1%)	1525 (8.9%)	355 (5.9%)	<0.001
**Anaemia in pregnancy**	211 (0.9%)	127 (0.7%)	84 (1.4%)	<0.001
**ICP**	203 (0.9%)	166 (1.0%)	37 (0.6%)	0.04
**Infectious disease screening**				
VDRL-positive	72 (0.3%)	20 (0.1%)	52 (0.9%)	<0.001
HBsAg-positive	231 (1.0%)	33 (0.2%)	198 (3.4%)	<0.001
HCV antibody-positive	100 (0.5%)	65 (0.4%)	35 (0.6%)	0.04
HIV antibody-positive	29 (0.1%)	16 (0.1%)	13 (0.2%)	0.02
GBS swab-positive	4713 (22.5%)	3538 (22.6%)	1175 (22.2%)	0.48
**Gestational age**				
Moderate to late preterm	1268 (5.5%)	927 (5.4%)	341 (5.7%)	0.38
Very preterm	156 (0.7%)	112 (0.7%)	44 (0.7%)	0.50
Extremely preterm	74 (0.3%)	48 (0.3%)	26 (0.4%)	0.07
**Stillbirth**	72 (0.3%)	54 (0.3%)	18 (0.3%)	0.88
**Mode of delivery**				
Vaginal delivery	16,383 (70.7%)	12,114 (70.5%)	4269 (71.3%)	0.26
Caesarean section	5527 (23.9%)	4115 (24.0%)	1412 (23.6%)	0.56
Assisted/operative vaginal delivery	1255 (5.4%)	948 (5.5%)	307 (5.1%)	0.25
**Induction** (* N = 23,100)	4323 (22.2%)	3169 (21.9%)	1154 (22.9%)	0.21
**Epidural analgesia**	1035 (4.5%)	808 (4.7%)	227 (3.8%)	0.003
**Episiotomy** (N = 12,110)	1792 (14.8%)	1363 (15.0%)	429 (14.2%)	0.27
**Neonatal outcomes**				
Low weight at birth (<2500 g)	1704 (7.2%)	1262 (7.2%)	442 (7.3%)	0.59
Weight at birth ≥ 4500 g	117 (0.5%)	82 (0.5%)	35 (0.6%)	0.88
5 min APGAR score ≤ 3	119 (0.5%)	83 (0.5%)	36 (0.6%)	0.26
Neonatal resuscitation with oxygen	941 (4.0%)	664 (3.8%)	277 (4.6%)	0.009
Neonatal resuscitation with intubation	161 (0.7%)	111 (0.6%)	50 (0.8%)	0.32
Phototherapy (* N = 23,097)	1039 (4.5%)	745 (4.4%)	294 (4.9%)	0.08
NICU admission (* N = 22,953)	2554 (11.1%)	1798 (10.5%)	756 (12.6%)	<0.001
Neonatal hospitalisation (days) (* N = 23,102)				0.50
Mean ± SD	4.2 ± 6.0	4.1 ± 5.7	4.3 ± 6.7	
Median (IQR)	3.0 (3.0–4.0)	3.0 (3.0–4.0)	3.0 (3.0–4.0)	
Formula feeding at discharge (* N = 22,627)	704 (3.1%)	600 (3.6%)	104 (1.8%)	<0.001

Statistical significance was assessed using the chi-square test or Fisher’s exact test, and the Wilcoxon–Mann–Whitney test for continuous variables. * N varies across variables due to missing data. Abbreviations: GDM, gestational diabetes mellitus; ICP, intrahepatic cholestasis of pregnancy; VDRL, Venereal Disease Research Laboratory test; HBsAg, hepatitis B surface antigen; HCV, hepatitis C virus; HIV, human immunodeficiency virus; GBS, Group B Streptococcus; NICU, neonatal intensive care unit.

**Table 3:** Adjusted odds ratios (aORs) of adverse pregnancy outcomes among women with foreign citizenship compared with Italian women, estimated using multivariable logistic regression models.

	aOR	95% CI	*p*-Value
Gestational hypertension *	0.77	0.54–1.11	0.16
Preeclampsia *	1.28	0.90–1.83	0.17
Gestational diabetes mellitus *	2.17	1.86–2.53	<0.001
Intrahepatic cholestasis of pregnancy *	0.65	0.43–0.98	0.04
GBS-positive	0.98	0.89–1.07	0.62
Moderate to late preterm **	0.95	0.72–1.26	0.07
Very preterm **	0.52	0.19–1.47	0.21
Extremely preterm **	2.03	1.10–3.75	0.02

All models adjusted for maternal age, marital status, educational level, parity, employment status and smoking during pregnancy. * Additionally adjusted for medically assisted reproduction, multiple pregnancy and maternal obesity. ** Additionally adjusted also for medically assisted reproduction, multiple pregnancy, hypertensive disorders in pregnancy, GDM, maternal obesity, placental disorders, uterine malformations, maternal infections, congenital anomalies and intrauterine growth retardation.

**Table 4:** Adjusted odds ratios (aORs) of adverse intrapartum outcomes among women with foreign citizenship compared with Italian women, estimated using multivariable logistic regression models.

	aOR	95% CI	*p*-Value
Caesarean section *	1.14	1.04–1.25	0.01
Assisted/operative vaginal delivery *	1.01	0.84–1.21	0.91
Induction **	1.05	0.95–1.16	0.31
Epidural analgesia °	0.92	0.77–1.10	0.34
Episiotomy °°	1.05	0.90–1.24	0.52

All models adjusted for maternal age, marital status, educational level, parity, employment status, multiple pregnancy and smoking during pregnancy. * Additionally adjusted for maternal and foetal conditions and foetal distress. ** Additionally adjusted for maternal and foetal conditions, foetal distress, gestational age > 41 weeks, medically assisted reproduction, mode of delivery and pre-labour rupture of membranes at term (PROM). ° Additionally adjusted for labour induction and oxytocin augmentation. °° Additionally adjusted for mode of delivery and epidural analgesia.

**Table 5:** Adjusted odds ratios (aORs) of neonatal outcomes among children born to women with foreign citizenship compared with Italian women, estimated using multivariable regression models.

	aOR/Coefficient	95% CI	*p*-Value
Birth weight < 2500 g *	1.10	0.95–1.28	0.21
Birth weight ≥ 4500 g **	1.19	0.74–1.92	0.47
5 min Apgar score ≤ 3 °	1.63	0.86–3.12	0.14
Neonatal resuscitation with oxygen °°	1.16	0.98–1.39	0.08
Phototherapy ^#^	1.23	1.04–1.47	0.02
NICU admission °°	1.33	1.17–1.51	<0.001
Formula feeding at discharge ^##^	0.39	0.30–0.50	<0.001
Length of neonatal hospitalisation (days) °°	β = 0.17	−0.03–0.38	0.10

All models adjusted for maternal age, marital status, educational level, parity, employment status, multiple pregnancy and smoking during pregnancy. * Additionally adjusted for placental disorders, PROM, and hypertensive disorders in pregnancy and maternal infections. ** Additionally adjusted for GDM. ° Additionally adjusted for congenital anomalies, intrauterine growth restriction, preterm birth and mode of delivery. °° Additionally adjusted for congenital anomalies, intrauterine growth restriction, preterm birth, mode of delivery and GDM/NICU. # Additionally adjusted for preterm birth and type of feeding. ## Additionally adjusted for preterm birth, mode of delivery, epidural analgesia, 5 min Apgar score, and attendance at antenatal classes. Abbreviations: NICU, Neonatal Intensive Care Unit; PROM, pre-labour rupture of membranes.

## Data Availability

The data underlying this article cannot be shared publicly due to privacy and ethical restrictions related to the use of sensitive health data. Access to the data is subject to approval by the competent authorities and is not freely available.

## Abbreviations

GDM	Gestational Diabetes Mellitus
A MatCH For Women	Access to Maternal and Child Health Services by Foreign Women
CedAP	Birth Assistance Certificate
APSS	Provincial Health Services
ISTAT	Italian National Institute of Statistics
EU	European Union
NICU	Neonatal Intensive Care Unit
GBS	Group B Streptococcus
ICP	Intrahepatic Cholestasis of Pregnancy
PROM	Pre-Labour Rupture of Membranes
VDRL	Venereal Disease Research Laboratory test
HCV	Hepatitis C Virus
HIV	Human Immunodeficiency Virus
HBV	Hepatitis B Virus
ICD-9-CM	International Classification of Diseases, Ninth Revision, Clinical Modification
SD	Standard Deviation
IQR	Interquartile Range
